# First report of the efficacy of a fluralaner-based pour-on product (Exzolt® 5%) against ectoparasites infesting cattle in Brazil

**DOI:** 10.1186/s13071-023-05934-7

**Published:** 2023-09-26

**Authors:** Alvimar José da Costa, João Ricardo de Souza Martins, Fernando de Almeida Borges, Luis Fernando Vettorato, Francisco Bonomi Barufi, Heitor de Oliveira Arriero Amaral, Luara Carolina Abujamra, Daniel de Castro Rodrigues, Welber Daniel Zanetti Lopes

**Affiliations:** 1 Faculdade de Ciências Agrárias e Veterinárias, Universidade Estadual Paulista, Jaboticabal, São Paulo Brazil; 2 Instituto de Pesquisas Desidério Finamor, Eldorado do Sul, Rio Grande do Sul Brazil; 3 Departamento de Medicina Veterinária, Universidade Federal do Mato Grosso Do Sul, Campo Grande, Mato Grosso do Sul Brazil; 4 MSD Saúde Animal, São Paulo, Brazil; 5 Centro de Parasitologia Veterinária, Universidade Federal de Goiás, Goiânia, Goiás Brazil

**Keywords:** *Cochliomyia hominivorax*, *Dermatobia hominis*, Fluralaner, *Haematobia irritans*, Isoxazoline, *Rhipicephalus microplus*

## Abstract

**Background:**

This study describes the effectiveness of a novel active pharmaceutical ingredient, fluralaner (isoxazoline class), against important ectoparasites infesting cattle in Brazil.

**Methods:**

A total of 13 studies involving a 5% fluralaner-based pour-on formulation (Exzolt 5%; further referred to as Exzolt) were conducted. Specifically, the effectiveness of this formulation was studied against *Rhipicephalus microplus* (6 studies), *Cochliomyia hominivorax* larvae (4 studies), *Dermatobia hominis* larvae (1 study) and *Haematobia irritans* flies (2 studies).

**Results:**

The therapeutic efficacy of Exzolt was found to exceed 98% at 4 days post treatment (DPT), while persistent efficacy (> 90% efficacy) against repeated infestations of *R. microplus* was observed for up to 79 DPT. In field studies, ≥ 98% therapeutic efficacy was demonstrated at all study sites by 7 DPT, and a persistent efficacy (> 90% efficacy) was observed for 42, 49 or 56 DPT. Exzolt prevented *C. hominivorax* eggs from developing to the larval stage, thus mitigating the development of myiasis in cattle naturally and artificially infested with this screworm. The efficacy of Exzolt against *D. hominis* larvae was 98% at 3 DPT, while persistent efficacy (> 90% effectiveness) was found to last for up to 70 DPT. Against *H. irritans*, Exzolt showed therapeutic efficacy (≥ 90%) within the first day of treatment at both study sites, while persistent efficacy (≥ 90%) was observed for 7 DPT at one site and for 21 DPT at the other site.

**Conclusions:**

Overall, the results from these studies confirm that Exzolt is therapeutically efficacious against the most important ectoparasites infesting cattle in Brazil. The novel active pharmaceutical ingredient, fluralaner, provides a new treatment option for farmers to control cattle ectoparasites, especially where there is resistance to other chemical classes. In addition, an effective control of ectoparasites will improve overall cattle health and well-being as well as production.

**Graphical Abstract:**

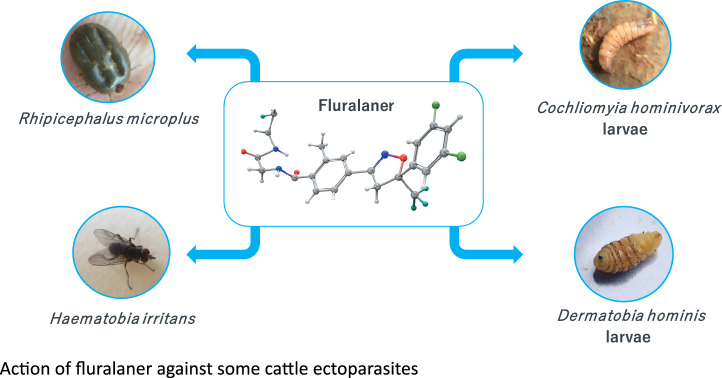

## Background

The control of cattle ectoparasites remains a major challenge for cattle farmers, particularly those in tropical and subtropical regions where infestations commonly cause irritation, stress, skin lesions and blood loss in animals and result in reduced productivity. There is also the potential of exposure to vector-borne pathogens that may cause disease [1–3] Ectoparasites of importance for cattle in Brazil include the cattle tick (*Rhipicephalus microplus*), horn fly (*Haematobia irritans*), cattle grub (*Dermatobia hominis*) and the New World screwworm (*Cochliomyia hominivorax*). The potential cost of these parasites to the Brazilian cattle industry is estimated to be about US$6.5 billion [4].

The significance of a cattle tick infestation may be twofold as both direct and potentially indirect effects can adversely affect the host. The direct effects of attachment and blood-feeding activities of the cattle tick can lead to tick-worry, severe skin lesions and anemia, causing significant reductions in animal growth and milk production [4]. In addition, there is the potential for indirect losses if the host is exposed to tick-borne organisms causing diseases such as babesiosis and anaplasmosis [1, 5, 6]. *Cochliomyia hominivorax* infestation is a listed disease by the World Organization for Animal Health [7] that infests warm-blooded animals (including humans), with cattle considered to be the primary host [8]. Screwworms lay eggs in the navels of neonates or in an animal’s wounds (e.g. insect bites, castration, shearing sores, de-horning wounds), which rapidly hatch to larvae. *Cochliomyia hominivorax* larvae then commence feeding and burrow deep into the wound, causing extensive tissue damage. Infestations can have devastating effects if myiasis develops and the infection is left untreated, leading to mortality of the host in severe cases [2]. *Dermatobia hominis* is a unique fly as it captures and deposits eggs on a vector (e.g. mosquito or small fly), which deposits the *D. hominis* eggs on the host [9] where it rapidly develops to the larval stage and penetrates the subcutaneous tissue of the host [10]. The larvae remain at the site of penetration and a nodule is formed surrounding the larvae, called ‘warble.’ The larvae will develop through three parasitic stages over a 5- to 6-week period, increasing in size and causing intense inflammation and pain before maturation and exiting through a perforation in the hide [9], resulting in significant damage. *Dermatobia hominis* infestations have also been indirectly associated with the disease “lechiguana” (focal proliferative fibrogranulomatous panniculitis) due to the transmission of *Mannheimia granulomatis*, leading to the development of large lesions; this disease can be fatal if left untreated [3]. *Haematobia irritans* is a blood-feeding ectoparasite and is considered to be one of the most important and costly parasites affecting cattle in Brazil [4]. The feeding behavior of this ectoparasite, continual fly pressure and the sheer numbers that may be present on cattle cause stress, irritation and substantial blood loss, resulting in decreased productivity [11, 12]. *Haematobia irritans* flies are also known to be a source of vector-borne diseases [4].

Despite many veterinarians and animal health consultants advocating the implementation of integrated parasite management strategies (pharmaceutical and non-pharmaceutical approaches to control parasites) as a means to control ectoparasites at cattle farms [4], the most common strategy for managing ectoparasites continues to be just the episodic administration of pharmaceutical products belonging to different chemical classes. The first ectoparasiticide demonstrating a broad-spectrum activity belonged to the class of “organochlorines,” which were used in the 1940s. Other classes of insecticides, such as the organophosphates, amidines and pyrethroids, were developed in the 1960s, 1970s and 1980s, respectively, followed by more recent classes of insecticides, the phenylpyrazoles and benzophenylureas, which became commercially available in the 1990s [13–17]. However, no new chemical class of chemicals with ectoparasiticidal activity has been discovered/commercially launched in the last 30 years or so.

In 2014, a new chemical class, the isoxazolines, were identified as possessing potent ectoparasiticidal activity against ectoparasites infesting dogs and cats [18]. Isoxazolines act by inhibiting the ɣ-aminobutyric acid (GABA)-gated chloride channels (GABACls) and l-glutamate-gated chloride channels (GluCls) within the central nervous system of ectoparasites, resulting in paralysis and death of the ectoparasite [19]. The first isoxazoline molecule developed was fluralaner [20, 21], later followed by sarolaner, afoxolaner and lotilaner. These active ingredients have only recently become available for the treatment of ectoparasites in dogs, cats and poultry [22–25]. Given the increasing prevalence of resistance of cattle ectoparasites to many of the pharmaceutical classes of insecticides [2], a gap in the market was identified and a novel pour-on formulation containing the active ingredient fluralaner (50 mg/ml) was developed that specifically targets the ectoparasites of cattle. In 2022, it this formulation was registered and approved for use in Brazil under the name Exzolt® 5% (referred to further as Exzolt).

This article is the first published report on Exzolt which summarizes the effectiveness of this formulation against four important ectoparasites (*R. microplus*, *C. hominivorax* larvae, *D. hominis* larvae and *H. irritans*) affecting cattle in Brazil, following its application as a pour-on solution to cattle at a single dose of 2.5 mg/kg body weight (BW).

## Methods

A total of 13 studies were conducted to assess the efficacy of Exzolt against *R. microplus* (6 studies), *C. hominivorax* larvae (4 studies), *D. hominis* larvae (1 study) and *H. irritans* flies (2 studies) in cattle at animal farms located in different regions of Brazil (Table 1). The study sites were located in several distinct geographical regions of the country, with the objective to assess the efficacy of Exzolt in cattle maintained under different climatic conditions. In all studies, only animals that had not received any ectoparasiticide treatment for at least 90 days prior to initiation of the studies were enrolled.

**Table 1:** Summary of the studies conducted to assess the effectiveness of Exzolt against ectoparasites on cattle from different regions in Brazil

Ectoparasite	Study number and infestation setting	Locality (city, state)	Breed type	Sex	Number of animals included (*n* per group)	Treatment group
*Rhipicephalus microplus* (cattle tick)	1. Experimental infestation	Itirapuã, São Paulo (southeast region)	Crossbred (Gyr × Holstein 7/8)	Male (all)	16 (*n* = 8/group)	T01: Exzolt T02: Negative control
	2. Natural infestation	São José do Rio Pardo, São Paulo (southeast region)	Simental	Female (non-lactating or pregnant)	20 (*n* = 10/group)	T01: Exzolt T02: Negative control
	3. Natural infestation	Formiga, Minas Gerais (southeast region)	Crossbred (Gyr × Holstein 7/8)	Male (all)	20 (*n* = 10/group)	T01: Exzolt T02: Negative control
	4. Natural infestation	Formiga, Minas Gerais (southeast region)	Crossbred (Gyr × Holstein 7/8)	Male (all)	20 (*n* = 10/group)	T01: Exzolt T02: Negative control
	5. Natural infestation	Ribas do Rio Pardo, Mato Grosso do Sul (central-west region)	Crossbred (Angus × Nelore 1/2)	Male (all)	20 (*n* = 10/group)	T01: Exzolt T02: Negative control
	6. Natural infestation	Santana do Livramento, Rio Grande do Sul (south region)	Angus	Males (all) and female (non-lactating or pregnant)	20 (*n* = 10/group)	T01: Exzolt T02: Negative control
*C. hominivorax* (New World screwworm) larvae	7. Preventative efficacy (natural infestation)	Rifaina, São Paulo (southeast region)	Crossbred (Gyr × Holstein 7/8)	Male (all) and female (non-lactating or pregnant)	12 (*n* = 6/group)	T01: Exzolt T02: Negative control
	8. Preventative efficacy (natural infestation)	Espírito Santo do Pinhal, São Paulo (southeast region)	Nelore	Male (all)	30 (*n* = 15/group)	T01: Exzolt T02: Negative control
	9. Curative efficacy	Formiga, Minas Gerais (southeast region)	Crossbred (Gyr × Holstein 7/8)	Male (all)	24 (*n* = 12/group)	T01: Exzolt T02: Negative control
	(natural infestation)					
	10. Curative efficacy (natural infestation)	Goiânia, Goiás (center-west region)	Crossbred (Gyr × Holstein 15/16)	Male (all) and female (non-lactating or pregnant)	12 (*n* = 6/group)	T01: Exzolt T02: Negative control
*Dermatobia hominis* (tropical warble fly) larvae	11. Natural infestation	São João da Boa Vista, São Paulo (southeast region)	Camchim (Charolês × Nelore 1/2)	Female (non-lactating or pregnant)	12 (*n* = 6/group)	T01: Exzolt T02: Negative control
*Haematobia irritans* (horn fly)	12. Natural infestation	Uberada, Minas Gerais (southeast region)	Crossbred (Angus × Nelore 1/2)	Female (non-lactating or pregnant)	30 (*n* = 15/group)	T01: Exzolt T02: Negative control
	13. Natural infestation	Santana do Livramento, Rio Grande do Sul (southern region)	Angus	Female (non-lactating or pregnant)	30 (*n* = 15/group)	T01: Exzolt T02: Negative control

In all studies (under either experimental or natural infestation setting), there were two treatment groups: treatment group T01, which comprised animals treated with Exzolt, and treatment group T02, which comprised animals treated with placebo. Throughout the study, animals from the two treatment groups were maintained in separate but similar spaces to prevent treatment cross-contamination. These spaces were pastures of similar sizes and quality and similar stocking rates in field studies and separate pens in the experimentally-induced infestation studies. Similarly, when cattle were brought into the cattle yards for inspection, treatment and ectoparasite counts, the two groups always remained separated.

All clinical procedures were performed in accordance with ‘Good Clinical Practices’ [26]. All studies were masked and all procedures using animals complied with the Ethical Principles in Animal Research, as adopted by the Brazilian Council of Animal Experimentation (CONCEA) and were approved by an Ethical Committee for Animal Welfare prior to the commencement of each study.

### Allocation of animals to treatment groups

In all studies, the animals were assigned to treatment groups in accordance with a randomized complete block design. Enrolled animals were ranked in the order of either their ectoparasite count or BW (dependent on the study, as detailed below), then blocked into blocks of two. Within each block, cattle were randomly allocated to one of two treatment groups: T01 (Exzolt) or T02 (negative control), such that each group had animals with similar mean and range of ectoparasite count or BW, depending on the type of study.

### Procedure for administration of test substances (Exzolt and placebo)

All animals were weighed prior to treatment and treated according to individual BW. Animals in the T01 group were treated with Exzolt at a dose rate of 2.5 mg fluralaner/kg BW (dose volume: 0.05 ml/kg BW, rounded up to the nearest milliliter). A disposable syringe was filled with the calculated amount of Exzolt for each animal, and the contents of the syringe were administered as a narrow strip on the back of the animal along the dorsal midline between the wither and tailhead. Animals in the T02 group were treated with a placebo formulation using a similar procedure and dose volume. The placebo formulation contained all of the excipients of the Exzolt formulation but without any fluralaner. At all study sites, animals were observed immediately and at approximately 1 h after treatment administration for the development of any adverse reaction. Thereafter, treatment administration sites were observed daily as a part of general health observations. A single treatment was performed in all studies.

### Assessment of efficacy of Exzolt against *R. microplus*

#### Assessment of efficacy under experimental infestation conditions (study 1)

Study 1 was conducted at a farm in Itirapuã, São Paulo State, Brazil. Sixteen tick-free, non-castrated crossbred animals between the age of 7 and 8 months, having a similar BW and body condition score, were enrolled in the study. On day 25 prior to treatment (D-25), the selected animals were placed in individual concrete block stalls (3 m^2^) in a covered barn, with a slatted floor to facilitate washing and tick collection. The animals had ad libitum access to water and were fed maize silage and a concentrated feed (1.5 kg/animal/day) once daily for the duration of the study. On D-25, each animal was infested with approximately 5000 *R. microplus* non-fed larvae (aged between 7 and 14 days); this artificial infestation process was continued on study days D-25, D-23, D-21, D-18, D-16, D-14, D-11, D-9, D-7, D-4 and D-2 [27, 28]. During the infestation process, cattle were tied up inside their stalls, and the tick larvae were delicately deposited along the animal’s dorsal line so that they were able to move inside the fur and select a fixation site. The animals were restrained for approximately 60 min post infestation. The *R. microplus* strain used for artificial challenge had been isolated and maintained on the farm where the study was conducted.

All fully engorged female ticks that naturally detached from each animal on D-3, D-2 and D-1 were collected and counted. The tick collection procedure involved flushing of the inside of each animal’s stall with water, conducted daily between 08:00 and 09:00 am. All detached ticks were captured in a wire mesh sieve bucket (aperture width: 4 mm) placed under the effluent outlet of each stall. After collection, the ticks were dried, cleaned and counted. Mean engorged female tick counts (between D-3 and D-1) were calculated for each individual animal, and cattle with a mean number of engorged female ticks > 20 were enrolled in the study. Animals were ranked based on each animal’s mean female tick count between D-3 to D-1 and blocked into blocks of two. Animals were then randomly allocated from within each block to one of the treatment groups, such that each treatment group had a similar mean and range of female tick count. Cattle were weighed and treated on D0 with their respective treatments. Treatment procedures were as described in “Procedure for administration of test substances (Exzolt and placebo)” section.

Following the treatment, all cattle were infested with approximately 5000 viable and unfed larvae twice weekly, commencing on D0 until the conclusion of the study on D90. For post-treatment tick counts, the slatted floors of individual pens were washed daily (between 8:00 to 9:00 a.m.) from D1 to D90, and any detached engorged female ticks were collected and counted.

Acaricidal efficacy of Exzolt was calculated considering the arithmetic means of tick counts, in accordance with previous recommendations [29, 30] and using the following formula as described in [27, 28, 31]:

$$\mathrm{Efficacy\, percentage}=\left[1-\frac{\mathrm{Ta} \times \mathrm{Cb}}{\mathrm{Tb} \times \mathrm{Ca}} \right] {\times }100,$$

where “Ta” is the mean number of engorged female ticks detached from treated animals after treatment administration; “Tb” is the mean number of engorged female ticks detached from animals over a period of 3 days prior to the treatment administration; “Ca” is the mean number of engorged female ticks detached from negative control animals after the treatment date; and “Cb” is the mean number of engorged female ticks detached from the negative control animals over a period of 3 days prior to the treatment administration.

Therapeutic efficacy was the assessment of the effect of Exzolt on ticks between D1 and D22 [28] or D23 [27] after treatment, and persistent efficacy was the evaluation of the protection period provided by Exzolt against new infestations following treatment administration on D0 [28].

#### Assessment of efficacy under natural infestation conditions (studies 2, 3, 4, 5 and 6)

The efficacy of Exzolt was assessed in animals naturally infested with *R. microplus* in five studies (studies 2–6). Twenty clinically healthy animals that were naturally infested with *R. microplus* were enrolled in each study. Animals were of similar breed, BW and body condition score and ranged between 8 and 18 months of age. Tick counts on cattle at each site were conducted on 3 consecutive days prior to treatment (D-3, D-2 and D-1), and only animals with mean engorged female tick (between 4.5 and 8.0 mm in length) counts > 20 were included in the studies. Animals were ranked based on each animal’s mean female tick count between D-3 and D-1 and blocked into blocks of two. Animals were then randomly allocated from within each block to one of the two treatment groups. Allocation was such that each treatment group had a similar arithmetic mean and range of female tick count. Cattle were weighed and treated on D0 with their respective treatments. Treatment procedures were as described in “Procedure for administration of test substances (Exzolt and placebo)” section.

To evaluate the efficacy of Exzolt, tick counts were performed on D3, D7, D14 and D21, and weekly thereafter until D56 (studies 3 and 4), D63 (studies 2 and 5) and D77 (study 6), in accordance with the technique described by Wharton and Utech [32]. Tick counts were performed on one side of each animal [28], and all ticks measuring 4.5–8.0 mm in length were counted [32]. Acaricidal efficacy was calculated based on arithmetic means, using the formula recommended by Roulston [31] and adopted by the Brazilian Ministry of Agriculture and Livestock [27]:

$$\mathrm{Efficacy \, percentage}=\left[1-\frac{\mathrm{Ta} \times \mathrm{Cb}}{\mathrm{Tb} \times \mathrm{Ca}} \right] {\times }100,$$

where “Ta” is the mean number of engorged female ticks detached from treated animals after treatment administration; “Tb” is the mean number of engorged female ticks detached from animals over a period of 3 days prior to the treatment administration; “Ca” is the mean number of engorged female ticks detached from negative control animals after the treatment date; and “Cb” is the mean number of engorged female ticks detached from the negative control animals over a period of 3 days prior to the treatment administration.

### Assessment of efficacy of Exzolt against *C. hominivorax*

#### Preventative efficacy (studies 7 and 8)

The preventative efficacy of Exzolt against the development of myiasis caused by *C. hominivorax* larvae was assessed in surgically created wounds that were subsequently subjected to natural infestation with *C. hominivorax* larvae [2]. In study 7, a wound (diameter: 4 cm) was surgically created through the infraspinous fossae and the dorsal border of the scapula of cattle, while in study 8 scrotal wounds were generated by surgical castration.

Study 7 was conducted in 12 animals [33] of approximately 13 months of age at an animal farm in Rifaina, São Paulo State. The cattle were allocated to treatment groups based on BW on D-8. The cattle were ranked in order of BW, blocked into blocks of two and then randomly allocated to one of the two treatment groups, such that each group had a similar group mean and range of BW. Treatments were administered on D-7 following the procedure described in “Procedure for administration of test substances (Exzolt and placebo)” section. Animals belonging to the two treatment groups were placed in separate paddocks to avoid treatment cross-contamination. For the wound creation, on D0, a 10-ml dose of local anesthetic (2% xylocaine; Anestésico Vansil®, Vansil Saúde Animal, Brazil) was administered in the infraspinous fossae and dorsal border of the scapula region on the left side of each animal, after which a cutaneous wound (diameter: 4 cm) was surgically created. The animals were returned to their respective grazing paddocks and exposed to natural infestations by *Cochliomyia hominivorax* larvae on the wounds.

The effectiveness of Exzolt in preventing myiasis caused by *C. hominivorax* larvae was evaluated by inspecting the lesions on each animal on D1, D2, D3, D4, D5, D6 and D7. Any myiasis caused by *C. hominivorax* larval infestation in the created wound was classified as active (at least 1 live *C. hominivorax* larvae per lesion); the wounds were also assessed for the presence of *C. hominivorax* egg mass.

The effectiveness of Exzolt in preventing myiasis caused by *C. hominivorax* larvae was calculated using the following formula [34, 35]:

$$\mathrm{Efficacy }({\%})= \frac{\text{Total number of animals with active larvae in CG}-\mathrm{Total of animals with active larvae in TG group}}{\text{Total of animals with active larvae in CG}}\times 100,$$

where “CG” refers to the placebo-treated control group and “TG” to the Exzolt-treated group.

The effectiveness of Exzolt in preventing myiasis against *C. hominivorax* larvae in surgically created castration wounds was evaluated in study 8 [34]. This study was conducted at a farm in Espírito Santo do Pinhal, São Paulo State, and 30 non-castrated male animals of approximately 18 months of age were enrolled in the study. Animals were weighed on D-1, ranked in order of BW, blocked into blocks of two and then randomly allocated to one of two treatment groups, such that each group had a similar group mean and range of BW. On D0, a 10-ml dose of local anesthetic (2% xylocaine) was administered subcutaneously into the distal region of the scrotum ("lid") of each animal. An experienced technician made an incision in the scrotum and removed the testicles from each animal. At the completion of each castration procedure, the treatments were administered following the procedure described in “Procedure for administration of test substances (Exzolt and placebo)” section, and animals belonging to each treatment group were then placed into separate grazing paddocks to avoid treatment cross-contamination. Over time, the surgical wounds would become infested naturally by *C. hominivorax* larvae. The effectiveness of Exzolt to prevent the development of *C. hominivorax* eggs to larvae (and development of myiasis) was evaluated daily for 14 days post treatment, and was calculated as described for study 7.

#### Therapeutic efficacy (studies 9 and 10)

Studies 9 and 10 were performed to assess the therapeutic efficacy of Exzolt against *C. hominivorax* larvae in naturally infested surgically created wounds.

Study 9 was conducted at a farm in Formiga, Minas Gerais State. Twenty-four non-castrated male cattle, aged approximately 15 months and weighing between 272 to 361 kg BW, were enrolled. On D-3, a 10-ml dose of local anesthetic (2% xylocaine) was administered in the infraspinous fossae and dorsal border of the scapula region on the left side of each animal after which a cutaneous wound (diameter: 4 cm) was surgically created [33, 36]. Cattle were then placed in a small grazing paddock so that infestations by *C. hominivorax* depositing egg masses on the wound and subsequent development of eggs to the larval stage could occur naturally.

Cattle were also weighed on the day of the wound induction and then allocated to treatment groups. Cattle were ranked in order of BW, blocked into blocks of two and then randomly allocated to one of two treatment groups, such that each group had a similar group mean and range of BW. After wound induction, the animals were examined daily to confirm the presence of egg masses and/or larvae of *C. hominivorax* in the wounds. Following confirmation that *C. hominivorax* larvae had established in the wound of each animal, cattle were then treated following the procedure described in “Procedure for administration of test substances (Exzolt and placebo)” section. Treatment was administered when third instar larvae were detected in each animal’s wound. After treatment administration the animals were placed in separate paddocks to avoid treatment cross-contamination.

The therapeutic efficacy of Exzolt was evaluated by inspecting the lesions of all cattle daily following treatment. Group T02 (negative control) animals were treated 3 days after myiasis was detected**.**

Study 10 was conducted at a farm in Goiânia, Goiás State, and followed the same methodology as described for study 9, with the exception that 12 animals (as compared to 24 in study 9) aged approximately 8 months and with a BW ranging from 99 to 211 kg were enrolled. In this study, the therapeutic efficacy of Exzolt was evaluated daily up to D7 post-treatment. The therapeutic efficacy of Exzolt for studies 9 and 10 was assessed using the same formula as used for studies 7 and 8.

### Assessment of efficacy of Exzolt against *D. hominis* larvae (study 11)

The efficacy of Exzolt was assessed in animals naturally infested with *D. hominis* in study 11. This study was conducted at a commercial farm in São João da Boa Vista, São Paulo State. Twelve female cattle aged approximately 36 months were enrolled in this study.

The number of larval nodules over the entire body of each animal was assessed by light compression (visual and tactile inspection) to determine whether they contained live *D. hominis* larvae [37]. Only animals with live *D. hominis* larvae counts > 10 were included in the study. Allocation of cattle to treatment groups was based on the number of live larval nodules on the body of each animal as assessed on D-1. Animals were ranked in order of number of live larval nodules, blocked into blocks of two and randomly allocated to one of two treatment groups. Cattle were weighed and treated on D0 in accordance with the treatment methodology described in “Procedure for administration of test substances (Exzolt and placebo)” section.

Live larval nodule counts on the body of each animal were performed on D3, D7, D14, D21, D28, D35, D42, D49, D56, D63, D70, D77 and D84 post-treatment, and the efficacy was calculated as [27, 28]:

$$\mathrm{Efficacy}= \frac{a-b}{a}\times 100,$$

where “*a*” is the mean number of live *D. hominis* larvae in the negative control group and “*b*” represents the mean number of live *D. hominis* larvae in the Exzolt group.

### Assessment of efficacy of Exzolt against *H. irritans* (studies 12 and 13)

Two studies were carried out (study 12 in Uberaba, Minas Gerais State and study 13 in Santa do Livramento, Rio Grande do Sul State) to assess the efficacy of Exzolt against *H. irritans* in naturally infested animals. In each of these studies, 30 female cattle, aged between 14 and 36 months of age were enrolled.

Animals were allocated to treatment groups based on the average *H. irritans* counts conducted on the whole-body surface of the animals on D-2 and D-1. All fly counts were simultaneously conducted by two trained technicians (one on the left and the other on the right side of the animal), between 07:00 and 10:00 a.m. at each time point [12]. Only animals with mean pre-treatment fly counts > 50 were included in the studies. Animals were ranked based on individual mean *H. irritans* counts, blocked into blocks of two and randomly allocated from each block to one of two treatment groups, such that each group had a similar group mean and range of *H. irritans* count. The same investigators performed counts on the same side of the cattle on all post-treatment dates.

Cattle were weighed and treated with their respective treatments on D0 following the treatment administration procedures described in “Procedure for administration of test substances (Exzolt and placebo)” section. Following treatment, cattle were placed in separate paddocks with a minimum distance of 5 km between each group to prevent any interference of the treatment on fly counts in the negative control group. After the administration of treatment, *H. irritans* counts were conducted on D1, D3, D7, and then weekly until D35 (study 13) and D49 (study 12),

To evaluate the efficacy of Exzolt against *H. irritans*, the following formula was used [27, 28]:

$$\mathrm{Efficacy}= \frac{a-b}{a}\times 100,$$

where “*a*” is the mean number of *H. irritans* in the negative control group and “*b*” represents the mean number of *H. irritans* in the Exzolt group.

### Statistical analysis

Data were analyzed using general linear models with SAS software version 9.4 [38]. *Rhipicephalus microplus*, *D. hominis* larvae and *H. irritans* counts were not normally distributed and were log transformed (count + 1) to ensure normality, homogeneity of variances, residual analysis and randomness of the observations with back-transformed least squares means. The effects included in the model were ectoparasite counts, treatment and time point. A protected Student t-test was used for mean separation and the significance level set to 5% (*P* < 0.05).

Analysis of data for viable *C. hominivorax* larvae and egg mass (independent of the viability) in wounds was conducted using SAS software version 9.4 [38], and Fisher's non-parametric test was used for mean separation, with the significance level set to 5% (*P* < 0.05).

## Results

### Study 1 (efficacy of Exzolt against *R. microplus* under the experimental infestation scenario)

This study assessed the therapeutic and persistent efficacy of Exzolt against artificial infestation and ongoing larval challenge of *R. microplus.* Assessment was conducted by the counting of engorged *R. microplus* female ticks which had detached from the animal and were collected each morning up until the completion of the study on D90. The therapeutic efficacy of 98.3% and 99.4% was observed on day 4 and 5 DPT, respectively and of > 99.9% between 6 and 22 DPT. On each of these occasions, the mean engorged female tick count in T01 (Exzolt) treatment group was significantly lower (*P* < 0.0001) than of cattle in T02 (negative control) (Table 2). The average therapeutic efficacy (95.1%) up to D22 and a persistent efficacy (> 90%) lasting until D70 were observed in this study.

**Table 2:** Mean fully engorged *Rhipicephalus microplus* female tick counts for each treatment group and efficacy of Exzolt over time from the artificial infestation study (Study 1)

Study 1: Southeast region—Itirapuã. São Paulo—Brazil							
Study day	Mean** fully engorged *R. microplus* counts				*P* value	Coefficient of variation	Efficacy (%)
	T01: Exzolt		T02: Negative Control				T01: Exzolt
D0*	77.58	A	73.33	A	0.9448	15.33	–
D1	46.25	A	80.00	A	0.1648	17.07	45.4
D2	26.50	B	88.50	A	0.0063	17.22	71.7
D3	13.50	B	95.25	A	0.0005	24.22	86.6
D4	1.50	B	84.63	A	< 0.0001	36.81	98.3
D5	0.50	B	83.25	A	< 0.0001	34.60	99.4
D6	0.00	B	86.13	A	< 0.0001	33.55	100.0
D7	0.00	B	96.63	A	< 0.0001	32.04	100.0
D8	0.00	B	83.38	A	< 0.0001	26.70	100.0
D9	0.00	B	87.50	A	< 0.0001	30.37	100.0
D10	0.00	B	80.38	A	< 0.0001	25.72	100.0
D11	0.00	B	71.63	A	< 0.0001	25.11	100.0
D12	0.00	B	67.50	A	< 0.0001	22.42	100.0
D13	0.00	B	62.38	A	< 0.0001	19.25	100.0
D14	0.00	B	60.63	A	< 0.0001	25.28	100.0
D15	0.00	B	69.88	A	< 0.0001	27.60	100.0
D16	0.00	B	74.25	A	< 0.0001	27.98	100.0
D17	0.00	B	73.63	A	< 0.0001	31.75	100.0
D18	0.00	B	80.38	A	< 0.0001	31.44	100.0
D19	0.00	B	72.38	A	< 0.0001	34.90	100.0
D20	0.00	B	64.88	A	< 0.0001	29.94	100.0
D21	0.00	B	65.13	A	< 0.0001	27.91	100.0
D22	0.00	B	58.25	A	< 0.0001	25.53	100.0
D23	0.00	B	54.75	A	< 0.0001	27.68	100.0
D24	0.00	B	55.75	A	< 0.0001	18.55	100.0
D25	0.00	B	55.00	A	< 0.0001	17.45	100.0
D26	0.00	B	57.13	A	< 0.0001	20.46	100.0
D27	0.00	B	55.25	A	< 0.0001	17.80	100.0
D28	0.00	B	51.75	A	< 0.0001	18.10	100.0
D29	0.00	B	55.13	A	< 0.0001	18.41	100.0
D30	0.00	B	51.75	A	< 0.0001	22.45	100.0
D31	0.00	B	50.50	A	< 0.0001	19.89	100.0
D32	0.00	B	56.38	A	< 0.0001	27.68	100.0
D33	0.00	B	57.38	A	< 0.0001	27.55	100.0
D34	0.13	B	63.50	A	< 0.0001	29.05	99.8
D35	0.00	B	65.63	A	< 0.0001	31.09	100.0
D36	0.00	B	79.13	A	< 0.0001	35.81	100.0
D37	0.00	B	86.00	A	< 0.0001	35.88	100.0
D38	0.25	B	102.25	A	< 0.0001	38.52	99.8
D39	0.00	B	107.13	A	< 0.0001	34.35	100.0
D40	0.00	B	105.63	A	< 0.0001	38.27	100.0
D41	0.00	B	112.88	A	< 0.0001	40.03	100.0
D42	0.00	B	117.50	A	< 0.0001	40.73	100.0
D43	0.00	B	108.50	A	< 0.0001	38.34	100.0
D44	0.00	B	109.38	A	< 0.0001	35.77	100.0
D45	0.00	B	111.50	A	< 0.0001	36.71	100.0
D46	0.00	B	123.88	A	< 0.0001	36.74	100.0
D47	0.25	B	117.00	A	< 0.0001	39.30	99.8
D48	0.00	B	124.63	A	< 0.0001	33.39	100.0
D49	0.00	B	131.25	A	< 0.0001	31.39	100.0
D50	0.00	B	136.63	A	< 0.0001	30.23	100.0
D51	0.25	B	135.50	A	< 0.0001	29.32	99.8
D52	0.00	B	122.88	A	< 0.0001	27.67	100.0
D53	0.00	B	131.88	A	< 0.0001	27.21	100.0
D54	0.00	B	133.50	A	< 0.0001	26.09	100.0
D55	0.00	B	137.13	A	< 0.0001	29.33	100.0
D56	0.00	B	142.38	A	< 0.0001	31.35	100.0
D57	0.00	B	140.63	A	< 0.0001	31.64	100.0
D58	0.00	B	148.88	A	< 0.0001	29.79	100.0
D59	0.00	B	146.50	A	< 0.0001	28.90	100.0
D60	0.88	B	123.75	A	< 0.0001	36.43	99.3
D61	0.75	B	117.63	A	< 0.0001	35.26	99.4
D62	1.50	B	104.75	A	< 0.0001	32.75	98.6
D63	0.88	B	107.00	A	< 0.0001	36.01	99.2
D64	0.88	B	100.75	A	< 0.0001	31.43	99.2
D65	1.25	B	85.75	A	< 0.0001	26.31	98.6
D66	1.75	B	91.88	A	< 0.0001	29.91	98.2
D67	2.88	B	89.38	A	< 0.0001	32.57	97.0
D68	1.13	B	83.00	A	< 0.0001	27.87	98.7
D69	1.63	B	85.00	A	< 0.0001	35.64	98.2
D70	2.00	B	82.13	A	< 0.0001	33.56	97.7
D71	1.25	B	76.88	A	< 0.0001	31.41	98.5
D72	1.25	B	80.13	A	< 0.0001	31.64	98.5
D73	2.38	B	72.25	A	< 0.0001	35.93	96.9
D74	3.14	B	75.88	A	< 0.0001	32.24	96.1
D75	5.71	B	85.38	A	< 0.0001	33.31	93.7
D76	6.14	B	86.00	A	< 0.0001	37.70	93.2
D77	6.00	B	84.50	A	0.0001	36.75	93.3
D78	7.57	B	88.63	A	0.0001	31.84	91.9
D79	9.00	B	91.25	A	0.0001	33.74	90.7
D80	10.00	B	88.63	A	0.0002	32.49	89.3
D81	7.71	B	96.13	A	0.0001	35.91	92.4
D82	10.43	B	100.63	A	0.0002	34.55	90.2
D83	11.43	B	102.88	A	0.0006	37.57	89.5
D84	11.43	B	104.38	A	0.0003	32.83	89.7
D85	10.43	B	109.00	A	0.0002	31.41	91.0
D86	14.00	B	104.00	A	0.0016	38.47	87.3
D87	14.25	B	94.50	A	0.0027	33.86	85.7
D88	24.00	B	112.13	A	0.0244	39.37	79.8
D89	27.00	B	116.38	A	0.0138	31.93	78.1
D90	24.63	B	110.88	A	0.0185	32.11	79.0
Efficacy for the entire period (D1 to D90)^3^	3.47	B	92.00	A	< 0.0001	22.02	96.4
Therapeutic efficacy (until D22)^4^	4.01	B	76.66	A	< 0.0001	20.22	95.1
Therapeutic efficacy (until D23)^5^	3.84	B	75.71	A	< 0.0001	20.33	95.2
Persistent efficacy (D23 up to D85)	1.91	B	96.13	A	< 0.0001	24.99	98.1

^*^ Mean tick counts before treatment (between D-3 and D-1)

^**^Means within rows followed by different capital letters are different at *P* < 0.05

Exzolt: Fluralaner 2.5 mg/kg BW

3: In accordance with EMEA (2021), an overall efficacy of more than 90% up to 100% is required

4: In accordance with WAAVP (2022), an efficacy at least 90% is required on the first 22 days post-treatment

5: In accordance with Brazil (1997), an efficacy at least 95% is required on the first 23 days post-treatment

### Studies 2–6 (efficacy of Exzolt against *R. microplus* under natural infestation scenario)

The therapeutic efficacy of Exzolt against field strains of *R. microplus* was assessed on days 3, 7, 14 and 21 post-treatment (D3, D7, D14 and D21). Ticks between 4.5 and 8.0 mm in length were counted on each occasion. The therapeutic efficacy was > 95% at all study sites. On D3, efficacy was < 90% at only two sites (studies 4 and 5). Tick counts continued on a weekly basis following D21 to determine the persistent efficacy, which was found to be D42 at three sites (studies 2, 3 and 4), D49 for study 5 and day 56 for study 6 (Table 3).

**Table 3:** Mean *R. microplus* female tick counts (length between 4.5 and 8.0 mm) for each treatment group and efficacy of Exzolt over time from the five field studies (studies 2–6)

Study 2: Southeast region—São José do Rio Pardo. São Paulo—Brazil							
Study day	Mean** *R. microplus* (4.5 to 8.0 mm length) counts				*P* value	Coefficient of variation	Efficacy (%)
	T01: Exzolt		T02: Negative Control				T01: Exzolt
D0*	31.33	A	31.57	A	0.9613	9.55	–
D3	3.10	B	32.50	A	< 0.0001	30.37	90.4
D7	0.70	B	34.80	A	< 0.0001	22.89	98.0
D14	0.20	B	28.50	A	< 0.0001	13.33	99.3
D21	0.00	B	32.50	A	< 0.0001	4.23	100.0
D28	0.00	B	34.50	A	< 0.0001	6.51	100.0
D35	0.00	B	34.60	A	< 0.0001	8.63	100.0
D42	0.70	B	23.00	A	< 0.0001	22.01	96.9
D49	8.30	B	22.10	A	0.0020	47.49	62.2
D56	10.30	B	24.90	A	0.0024	35.27	58.3
D63	28.60	A	27.70	A	0.1215	23.99	0.0
Therapeutic efficacy (until D21)	1.00	B	32.08	A	< 0.0001	17.43	96.9
Persistent efficacy (D28 up to D49)	2.25	B	28.55	A	< 0.0001	30.93	92.1
Mean efficacy between D7 and D14	0.45	B	31.65	A	< 0.0001	16.84	98.6

Study 3: Southeast region—Formiga. Minas Gerais—Brazil							
Study day	Mean** *R. microplus* (4.5 to 8.0 mm length) counts				p Value	Coefficient of variation	Efficacy (%)
	T01: Exzolt		T02: Negative Control				T01: Exzolt
D0*	38.13	A	38.07	A	0.9653	13.13	–
D3	2.20	B	35.10	A	< 0.0001	26.28	93.7
D7	0.00	B	41.90	A	< 0.0001	15.51	100.0
D14	0.00	B	47.90	A	< 0.0001	18.52	100.0
D21	0.00	B	63.40	A	< 0.0001	18.82	100.0
D28	0.00	B	66.00	A	< 0.0001	14.58	100.0
D35	0.10	B	63.40	A	< 0.0001	15.39	99.8
D42	2.00	B	66.50	A	< 0.0001	23.95	97.0
D49	26.90	B	67.00	A	0.0053	23.27	59.9
D56	56.70	A	73.10	A	0.1516	12.32	22.6
Therapeutic efficacy (until D21)	0.55	B	47.08	A	< 0.0001	19.53	98.8
Persistent efficacy (D28 up to D42)	0.70	B	65.30	A	< 0.0001	17.67	98.9
Mean efficacy between D7 and D14	0.00	B	44.90	A	< 0.0001	15.80	100.0

Study 4: Southeast region—Formiga. Minas Gerais—Brazil							
Study day	Mean** *R. microplus* (4.5 to 8.0 mm length) counts				*P* value	Coefficient of variation	Efficacy (%)
	T01: Exzolt		T02: Negative Control				T01: Exzolt
D0*	34.70	A	34.93	A	0.9526	12.26	–
D3	4.50	B	34.70	A	< 0.0001	32.19	86.9
D7	0.00	B	35.20	A	< 0.0001	18.72	100.0
D14	0.00	B	37.20	A	< 0.0001	18.41	100.0
D21	0.00	B	33.50	A	< 0.0001	17.72	100.0
D28	0.00	B	36.20	A	< 0.0001	16.92	100.0
D35	0.00	B	38.70	A	< 0.0001	15.49	100.0
D42	3.30	B	36.00	A	< 0.0001	32.27	90.8
D49	9.00	B	39.40	A	< 0.0001	18.65	77.0
D56	37.20	A	47.10	A	0.1422	12.88	20.5
Therapeutic efficacy (until D21)	1.13	B	35.15	A	< 0.0001	19.13	96.8
Persistent efficacy (D28 up to D49)	3.08	B	37.58	A	< 0.0001	19.90	91.8
Mean efficacy between D7 and D14	0.00	B	36.20	A	< 0.0001	18.58	100.0

Study 5: Center-West region—Ribas do Rio Pardo. Mato Grosso do Sul—Brazil							
Study day	Mean** *R. microplus* (4.5 to 8.0 mm length) counts				p Value	Coefficient of variation	Efficacy (%)
	T01: Exzolt		T02: Negative Control				T01: Exzolt
D0*	24.93	A	25.27	A	0.8758	5.95	–
D3	3.00	B	12.40	A	0.0003	39.33	75.5
D7	0.00	B	17.70	A	< 0.0001	13.65	100.0
D14	0.00	B	19.10	A	< 0.0001	11.96	100.0
D21	0.00	B	20.90	A	< 0.0001	32.39	100.0
D28	0.00	B	23.10	A	< 0.0001	11.70	100.0
D35	0.00	B	19.20	A	< 0.0001	12.80	100.0
D42	0.00	B	28.10	A	< 0.0001	16.56	100.0
D49	0.20	B	19.70	A	< 0.0001	25.82	99.0
D56	12.30	B	20.20	A	0.0063	15.88	38.3
D63	16.10	B	25.60	A	0.0174	16.05	36.3
Therapeutic efficacy (until D21)	0.75	B	17.53	A	< 0.0001	11.51	95.7
Persistent efficacy (D28 up to D49)	0.05	B	22.53	A	< 0.0001	12.47	99.8
Mean efficacy between D7 and D14	0.00	B	18.40	A	< 0.0001	6.04	100.0

Study 6: South region—Santana do Livramento. Rio Grande do Sul—Brazil							
Study day	Mean** *R. microplus* (4.5 to 8.0 mm length) counts				p Value	Coefficient of variation	Efficacy (%)
	T01: Exzolt		T02: Negative Control				T01: Exzolt
D0*	40.53	A	39.17	A	0.8978	15.47	–
D3	0.00	B	19.20	A	< 0.0001	27.70	100.0
D7	0.00	B	25.80	A	< 0.0001	20.58	100.0
D14	0.00	B	16.20	A	< 0.0001	37.04	100.0
D21	0.00	B	24.40	A	< 0.0001	61.63	100.0
D28	0.00	B	36.80	A	< 0.0001	45.03	100.0
D35	0.00	B	83.90	A	< 0.0001	9.49	100.0
D42	0.00	B	64.20	A	< 0.0001	8.13	100.0
D49	0.00	B	31.90	A	< 0.0001	25.35	100.0
D56	0.00	B	19.40	A	< 0.0001	18.79	100.0
D63	3.10	B	13.40	A	0.0001	35.43	77.6
D70	8.60	B	18.80	A	0.0137	24.74	55.8
D77	15.30	B	25.50	A	0.0297	20.33	42.0
Therapeutic efficacy (until D21)	0.00	B	21.40	A	< 0.0001	26.33	100.0
Persistent efficacy (D28 up to D70)	1.67	B	38.34	A	< 0.0001	15.73	95.8
Mean efficacy between D7 and D14	0.00	B	21.00	A	< 0.0001	23.43	100.0

Exzolt: Fluralaner 2.5 mg/kg body weight

^a^The respective preventative treatment was administered on D0. D1 to D77 are days post-treatment

^b^Mean tick counts were determined before treatment (between 3 and 1 days pre-treatment [D-3 and D-1]). Means within rows followed by different uppercase letters are significantly different at *P* < 0.05

### Studies 7 and 8 (preventative efficacy of Exzolt against *C. hominivorax*-induced myiasis)

The effectiveness of Exzolt in preventing *C. hominivorax* egg masses developing to the larval stage and causing active myiasis is detailed in Tables 4 and 5. In study 7, treatments were administered 7 days prior to the creation of the cutaneous wound in the infraspinous fossae and dorsal border of the scapula region on the left side of each animal, while in study 8 the creation of the scrotal wound and treatment occurred concurrently with treatment on the same day. The number of animals presenting egg masses deposited in the artificially created wounds was similar for both groups (*P* > 0.05) in each study, confirming that animals from both groups were challenged similarly by *C. hominivorax* in each study. Treatment with Exzolt prevented *C. hominivorax* eggs developing to the larval stage and causing myiasis in all animals in the T01 treatment group in both studies, while active myiasis was observed in all animals belonging to the negative control groups (T02).

**Table 4:** Number of animals in each group having egg masses or active myiasis caused by *Cochliomyia hominivorax* larvae in induced wounds and the preventative efficacy of Exzolt over time (study 7)

Observation (presence)	Study day	Total** number of animals with *C. hominivorax* egg mass or live larvae in induced wounds in each group				Efficacy (%)
		T01: Exzolt		T02: Negative Control		T01: Exzolt
Study 7: Southeast region—Rifaina. São Paulo—Brazil						
Egg mass presence	D0*	3	A	2	A	Not applicable
	D1	5	A	4	A	
	D2	4	A	6	A	
	D3	4	A	6	A	
	D4	4	A	4	A	
	D5	4	A	4	A	
	D6	4	A	3	A	
	D7	4	A	3	A	
Active *C. hominivorax* larvae presence	D0*	0	A	0	A	–
	D1	0	A	0	A	–
	D2	0	A	3	A	100.0
	D3	0	B	5	A	100.0
	D4	0	B	6	A	100.0
	D5	0	B	6	A	100.0
	D6	0	B	5	A	100.0
	D7	0	B	6	A	100.0

Exzolt: Fluralaner 2.5 mg/kg boy weight

^a^The respective preventative treatment was administered on D-7, and the wounds were induced on D0

^b^Total number of animals with *C. hominivorax* egg mass or live larvae in induced wounds. Means within rows followed by different uppercase letters are significantly different at *P* < 0.05

**Table 5:** Number of animals in each group having egg masses or active myiasis caused by *C. hominivorax* larvae in surgically castrated males and the preventative efficacy of Exzolt over time (study 8)

Observation (presence)	Study day	Total** number of animals with *C. hominivorax* egg mass or live larvae in scrotal wounds				Efficacy (%)
		T01: Exzolt		T02: Negative Control		T01: Exzolt
Study 8: Southeast region—Espírito Santo do Pinhal. São Paulo—Brazil						
Egg mass presence	D0*	–		–		Not applicable
	D1	0	A	0	A	
	D2	3	A	3	A	
	D3	0	A	3	A	
	D4	3	A	8	A	
	D5	0	A	1	A	
	D6	4	A	0	A	
	D7	5	A	5	A	
	D8	3	A	2	A	
	D9	0	A	0	A	
	D10	2	A	0	A	
	D11	1	A	1	A	
	D12	1	A	0	A	
	D13	0	A	0	A	
	D14	0	A	0	A	
Active *C. hominivorax* larvae presence	D0*	–		–		–
	D1	0	A	0	A	–
	D2	0	A	0	A	–
	D3	0	A	3	A	100.0
	D4	0	B	6	A	100.0
	D5	0	B	11	A	100.0
	D6	0	B	11	A	100.0
	D7	0	B	11	A	100.0
	D8	0	B	11	A	100.0
	D9	0	B	11	A	100.0
	D10	0	B	11	A	100.0
	D11	0	B	11	A	100.0
	D12	0	B	11	A	100.0
	D13	0	B	10	A	100.0
	D14	0	B	8	A	100.0

Exzolt: Fluralaner 2.5 mg/kg BW

^a^The orchiectomy procedures and the respective preventative treatment was administered simultaneously on D0

^b^Total number of animals with *C. hominivorax* egg mass or live larvae in induced wounds. Means within rows followed by different uppercase letters are significantly different at *P* < 0.05

### Studies 9 and 10 (therapeutic efficacy of Exzolt against *C. hominivorax* induced myiasis):

The results of therapeutic efficacy of Exzolt against active myiasis caused by *C. hominivorax* larvae are presented in Table 6. In both studies, the development of *C. hominivorax* myiasis was confirmed on D0 prior to the treatment administration on the same day. On D1, there was a significant reduction (*P* < 0.05) in the number of animals demonstrating active myiasis in the Exzolt groups in both studies, and by D3 there were no myiasis observed on any animal treated with Exzolt in either study. In contrast, active myiasis persisted in all animals belonging to the negative control group in both studies.

**Table 6:** Number of animals in each group having egg masses or active myiasis caused by *C. hominivorax* larvae in artificially induced wounds and the efficacy of Exzolt over time (studies 9 and 10)

Observation (presence)	Study day	Total** number of animals with *C. hominivorax* egg mass or live larvae in induced wounds				Efficacy (%)
		T01: Exzolt		T02: Negative Control		T01: Exzolt
*Study 9: Southeast region—Formiga. Minas Gerais—Brazil*						
Egg mass presence	Treatment day	8	A	5	A	Not applied
	1DPT	7	A	3	A	
	2DPT	4	A	1	A	
	3DPT	5	A	0	B	
Active *C. hominivorax* larvae presence	Treatment day	11	A	11	A	–
	1DPT	2	B	11	A	81.8
	2DPT	1	B	11	A	90.9
	3DPT	0	B	11	A	100.0
*Study 10: Center-West region—Goiânia. Goiás—Brazil*						
Egg mass presence	D0*	1	A	3	A	Not applied
	D1	3	A	2	A	
	D2	1	A	5	A	
	D3	1	A	5	A	
	D4	3	A	3	A	
	D5	2	A	2	A	
	D6	2	A	2	A	
	D7	2	A	0	A	
Active *C. hominivorax* larvae presence	D0*	6	A	6	A	–
	D1	2	B	6	A	66.7
	D2	2	B	6	A	66.7
	D3	0	B	6	A	100.0
	D4	0	B	6	A	100.0
	D5	0	B	6	A	100.0
	D6	0	B	6	A	100.0
	D7	0	B	6	A	100.0

Exzolt: Fluralaner 2.5 mg/kg body weight

*DPT* Days post-treatment

^a^Wound induction occurred on day 3 pre-treatment (D-3) and the respective preventative treatment was administered after randomization on D0

^b^Total number of animals with *C. hominivorax* egg mass or live larvae in induced wounds. Means within rows followed by different uppercase letters are significantly different at *P* < 0.05

### Study 11 (effectiveness of Exzolt against *D. hominis* larvae):

Animals enrolled in this study had confirmed natural infestations of *D. hominis* larvae prior to the respective treatment administration on D0. There was a significant reduction (*P* ≤ 0.05) in *D. hominis* larval counts in animals treated with Exzolt from D1 (73.1%). Efficacy increased to 97.7% by D3 and from D7 to D49, no larvae were found on any animal treated with Exzolt, whilst counts in the negative control group (T02) remained constant throughout the study period and were significantly higher (*P* < 0.0001) than cattle in T01. The persistent efficacy for Exzolt remained above the 90% threshold up to Day 70 (93.7%), and by Day 84, efficacy declined to 70.9%. Results are presented in Table 7.

**Table 7:** Mean *Dermatobia hominis* larvae counts for each treatment group and efficacy of Exzolt over time (study 11)

Study 11: Região Sudeste—São João da Boa Vista. São Paulo—Brazil							
Study day	Mean* counts of *Dermatobia hominis* larvae				p Value	Coefficient of variation	Efficacy (%)
	T01: Exzolt		T02: Negative Control				T01: Exzolt
D-1	21.60	A	21.50	A	0.9893	16.39	–
D1	5.90	B	21.90	A	0.0001	18.11	73.1
D3	0.50	B	21.70	A	0.0001	28.99	97.7
D7	0.00	B	21.80	A	< 0.0001	22.03	100.0
D14	0.00	B	20.90	A	< 0.0001	20.36	100.0
D21	0.00	B	20.50	A	< 0.0001	20.79	100.0
D28	0.00	B	20.00	A	< 0.0001	19.82	100.0
D35	0.00	B	19.50	A	< 0.0001	21.66	100.0
D42	0.00	B	16.70	A	< 0.0001	20.07	100.0
D49	0.00	B	16.40	A	< 0.0001	19.86	100.0
D56	0.10	B	15.50	A	< 0.0001	24.55	99.4
D63	0.50	B	10.10	A	0.0001	41.8	95.0
D70	0.70	B	11.50	A	0.0001	32.01	93.9
D77	2.60	B	13.60	A	0.0001	40.16	80.9
D84	4.40	B	15.10	A	0.0014	33.84	70.9

Exzolt: Fluralaner 2.5 mg/kg BW

^a^The respective preventative treatment was administered on D0

^b^Means within rows followed by different uppercase letters are significantly different at *P* < 0.05

### Studies 12 and 13 (effectiveness of Exzolt against *H. irritans*)

Animals with a pre-existing and natural *H. irritans* infestation were enrolled in these studies. Following treatment with Exzolt on D0, *H. irritans* counts were conducted on D1, D3 and D7 and weekly thereafter until D49 and D35 for studies 12 and 13, respectively (Table 8). Therapeutic efficacy > 90% for the Exzolt group was observed between D1 to D7 in study 12 and between D1 to D21 in study 13.

**Table 8:** Mean *Haematobia irritans* counts for each treatment group and efficacy of Exzolt over time (studies 12 and 13)

Study day	Mean** *Haematobia irritans* counts				p Value	Coefficient of variation	Efficacy (%)
	T01: Exzolt		T02: Negative Control				
*Study 12: Southeast region—Uberaba. Minas Gerais—Brazil*							
D0*	112.11	A	111.40	A	0.9496	5.70	–
D1	2.33	B	88.83	A	< 0.0001	27.24	97.4
D3	6.22	B	87.15	A	< 0.0001	12.10	92.9
D7	8.70	B	91.52	A	< 0.0001	15.22	90.5
D14	14.98	B	83.18	A	< 0.0001	15.28	82.0
D21	26.41	B	89.23	A	< 0.0001	9.80	70.4
D28	27.20	B	90.47	A	< 0.0001	9.96	69.9
D35	52.03	B	97.38	A	0.0004	9.83	46.6
D42	59.56	B	94.69	A	0.0052	9.54	37.1
D49	67.10	B	98.51	A	0.0113	8.68	31.9
*Study 13: South region—Santana do Livramento. Rio Grando do Sul—Brazil*							
D0*	162.07	A	163.40	A	0.9781	10.20	–
D1	1.20	B	177.87	A	< 0.0001	22.97	99.3
D3	1.47	B	120.80	A	< 0.0001	24.83	98.8
D7	0.93	B	107.33	A	< 0.0001	22.50	99.1
D14	0.53	B	100.13	A	< 0.0001	17.71	99.5
D21	4.67	B	84.13	A	< 0.0001	34.61	94.5
D28	60.27	B	140.67	A	< 0.0001	9.59	57.2
D35	50.67	B	140.13	A	< 0.0001	11.21	63.8

Exzolt: Fluralaner 2.5 mg/kg body weight

^a^The respective preventative treatment was administered on D0. Mean *H. irritans* counts on D0 before treatment (on D-2 and D-1)

^b^Means within rows followed by different capital letters are significantly different at *P* < 0.05

## Discussion

The studies reported here were designed taking into account both national [27] and international [28] guidelines to demonstrate therapeutic and/or persistent efficacy of Exzolt against the main ectoparasites of cattle (*R. microplus*,* C. hominivorax* larvae, *D. hominis* larvae and *H. irritans* flies) in Brazil.

In the experimental infestation study for *R. microplus,* cattle were housed in individual covered pens and not exposed to environmental elements (e.g. ultraviolet [UV] light, rain) during the study as these factors could impact the observed efficacy in field trials. One of the advantages of conducting an artificial challenge study is the elimination of a number of environmental factors, thereby establishing a confirmatory timing/duration for therapeutic and persistent efficacy [39, 40]. The results of this study demonstrated that > 90% efficacy was attained by day 4 post-treatment (D4) and efficacy remained above this threshold against ongoing tick larval challenge up to and including D79. In the field studies, the therapeutic efficacy of > 90% against natural *R. microplus* infestations was achieved by D7 and a persistent efficacy (> 90%) against continuous natural tick larval challenge lasted up to D42. The period of protection did differ slightly between sites, with an efficacy of > 99.9% reported on D56 at one site. Such differences could be due to environmental factors, such as UV light, humidity, rainfall and tick burden, which could vary at each site and could contribute to differences observed in the persistent efficacy [1, 40, 41]. The rapid onset of therapeutic efficacy together with the long period of persistent efficacy provided by Exzolt against *R. microplus* provide farmers with the opportunity to implement strategic cattle tick control programs [42–46], as well as with a reduced reliance on other pharmaceutical treatments against which ticks have already developed resistance, thereby ensuring the movement of ‘tick-free’ cattle across borders [28].

There are reports of increased prevalence of *R. microplus* populations that are resistant to different chemical classes of insecticides, such as the amidines, macrocyclic lactones, phenylpyrazoles and benzophenylurea [5, 47–55]. It is apparent from the published literature that *R. microplus* populations in those regions where studies with Exzolt were conducted were resistant to amitraz (250 ppm) [56, 57], abamectin and ivermectin 500 µg/kg BW pour-on, ivermectin 200 and 630 µg/kg BW injectable [58–60], fipronil 1 mg/mg pour-on [37], fluazuron 1.6 mg/kg + ivermectin 630 µg/kg (injectable) and diflubenzuron with 17 g of product added per kilogram of mineral salt [61, 62]. The studies reported here may indicate the effectiveness of Exzolt in ticks resistant to above-mentioned parasiticides.

*Cochliomyia hominivorax* is considered to be the main cause of myiasis in cattle in Brazil, with the umbilical region of neonates as a common site for infestation [8, 35], although infestations may occur in any type of wound. *Cochliomyia hominivorax* larvae differ from many other larval fly species in terms of their feeding habits as they ingest fresh rather than dead tissue [63]. This parasite causes a decline in productivity, and when infested wounds are left untreated, mortality is a likely outcome [4]. The most common strategy for prevention or treatment of myiasis is to administer an endectocide; however, this approach is not always successful. Eradication of *C. hominivorax* by using the sterile insect technique was a strategy implemented many years ago and was successful in North and Central America, as well as a number of other countries during the 1990s. However, South America continues to report cases of myiasis in cattle caused by *C. hominivorax* [63], and this will likely increase due to climate change. Our results show Exzolt to be an important and effective management tool for farmers to both prevent and treat myiasis caused by *C. hominivorax*, confirming that Exzolt prevented *C. hominivorax* egg masses from hatching to the larval stage and the subsequent development of the myiasis. In addition, Exzolt was shown to be a curative when applied to animals with an active *C. hominivorax* larval infestation.

Although the main impact of *D. hominis* infestation is production loss due to reduced gain in BW, the damage occurring to the cattle hide is of great concern, as leather is the second largest commodity exported by the Brazilian cattle industry [9]. Once the *D. hominis* larval stage has penetrated the subcutaneous tissue of the host, a nodule is formed that contains the larval ‘warble’ [10]. The warble increases in size during larval development stages while the larva is still contained within the nodule in the subcutaneous tissue. During this time, the hide is perforated, which allows the ‘warble’ to breath. This perforation also facilitates the discharge of exudate from the site of the active infestation. *Dermatobia hominis* larvae complete their life-cycle within 33–42 days and leave the host through the perforated hole in the hide. The damage caused by *D. hominis* larvae to the hide significantly decreases the commercial value of the leather [9]. Macrocyclic lactones have commonly been used to treat and prevent *D. hominis* larval infestations in cattle, but recent reports of resistance to this class of chemicals have been published [9, 64], thereby putting more pressure on farmers to consider alternative methods to control *D. hominis* larvae infestations. In the present study, Exzolt demonstrated a therapeutic efficacy of 97.7% within 3 days after treatment, and a persistent efficacy of > 90% was observed for up to 70 days post-treatment, as evidenced by the prevention, development and establishment of *D. hominis* to the larval stage and formation of nodules. Exzolt provides farmers with a very effective option to control *D. hominis*.

*Haematobia irritans* is one of the most important and costly parasites affecting cattle in Brazil, causing BW losses and stunted growth [4, 12]. Unfortunately, there is widespread resistance of *H. irritans* to the commonly used parasiticides (e.g. pyrethroids and organophosphates). The over-use of these treatments along with the high reproductive potential of *H. irritans* are just a number of the factors contributing to the development of resistance of *H. irritans*. In the present study, Exzolt demonstrated effectiveness against *H. irritans* infestations; however, its efficacy (> 90%) was variable from site to site, ranging between D7 at one site and D21 at the other (Table 8).

## Conclusion

This is the first report of the effectiveness of a new pour-on ectoparasiticide, Exzolt 5%, approved by Ministry of Agriculture, Livestock and Development (MAPA) in 2022. This report presents significant efficacy data of the product against common ectoparasites infesting cattle in Brazil, following its single administration at a dose of 2.5 mg/kg BW. The studies reported here demonstrate both the therapeutic and persistent efficacy against *Rhipicephalus microplus*, *Cochliomyia hominivorax* and *Dermatobia hominis* larvae and *Haematobia irritans*. Exzolt effectively controlled *R. microplus* burden in cattle for at least 42 days; it also effectively prevented and treated active myiasis caused by *C. hominivorax* larvae. Similarly, Exzolt was effective in the control of *D. hominis* larval ‘warbles’ and prevented (> 90% efficacy) re-infestation of *D. hominis* larvae for 70 days post-treatment. Exzolt also significantly reduced (> 90% efficacy) *H. irritans* burdens on cattle for at least 7 days post-treatment. Outcomes from these studies confirm that Exzolt 5% is very effective in controlling the most important ectoparasites infesting Brazilian cattle. Therefore, this product will be an important ectoparasite management tool for farmers to be included in their control programs when targeting or preventing ectoparasite infestations.

## Data Availability

Data used for the main conclusions must be in the manuscript, figures and tables or in a repository.
